# Two Time Point MS Lesion Segmentation in Brain MRI: An Expectation-Maximization Framework

**DOI:** 10.3389/fnins.2016.00576

**Published:** 2016-12-19

**Authors:** Saurabh Jain, Annemie Ribbens, Diana M. Sima, Melissa Cambron, Jacques De Keyser, Chenyu Wang, Michael H. Barnett, Sabine Van Huffel, Frederik Maes, Dirk Smeets

**Affiliations:** ^1^icometrixLeuven, Belgium; ^2^STADIUS Center for Dynamical Systems, Signal Processing and Data Analytics, Department of Electrical Engineering (ESAT), KU LeuvenLeuven, Belgium; ^3^Department of Neurology, Universitair Ziekenhuis Brussel, Vrije Universiteit Brussel (VUB)Brussel, Belgium; ^4^Department of Neurology, University Medical Center Groningen (UMCG)Groningen, Netherlands; ^5^Sydney Neuroimaging Analysis Centre, Brain and Mind Centre, University of SydneySydney, NSW, Australia; ^6^Medical Image Computing, Processing Speech and Images (PSI), Department of Electrical Engineering (ESAT), KU LeuvenLeuven, Belgium; ^7^ImecLeuven, Belgium; ^8^BioImaging Lab, Universiteit AntwerpenAntwerp, Belgium

**Keywords:** MSmetrix, multiple sclerosis, longitudinal lesion segmentation, expectation-maximization, MRI

## Abstract

**Purpose:** Lesion volume is a meaningful measure in multiple sclerosis (MS) prognosis. Manual lesion segmentation for computing volume in a single or multiple time points is time consuming and suffers from intra and inter-observer variability.

**Methods:** In this paper, we present MSmetrix-long: a joint expectation-maximization (EM) framework for two time point white matter (WM) lesion segmentation. MSmetrix-long takes as input a 3D T1-weighted and a 3D FLAIR MR image and segments lesions in three steps: (1) cross-sectional lesion segmentation of the two time points; (2) creation of difference image, which is used to model the lesion evolution; (3) a joint EM lesion segmentation framework that uses output of step (1) and step (2) to provide the final lesion segmentation. The accuracy (Dice score) and reproducibility (absolute lesion volume difference) of MSmetrix-long is evaluated using two datasets.

**Results:** On the first dataset, the median Dice score between MSmetrix-long and expert lesion segmentation was 0.63 and the Pearson correlation coefficient (PCC) was equal to 0.96. On the second dataset, the median absolute volume difference was 0.11 ml.

**Conclusions:** MSmetrix-long is accurate and consistent in segmenting MS lesions. Also, MSmetrix-long compares favorably with the publicly available longitudinal MS lesion segmentation algorithm of Lesion Segmentation Toolbox.

## 1. Introduction

**Figure 1:**
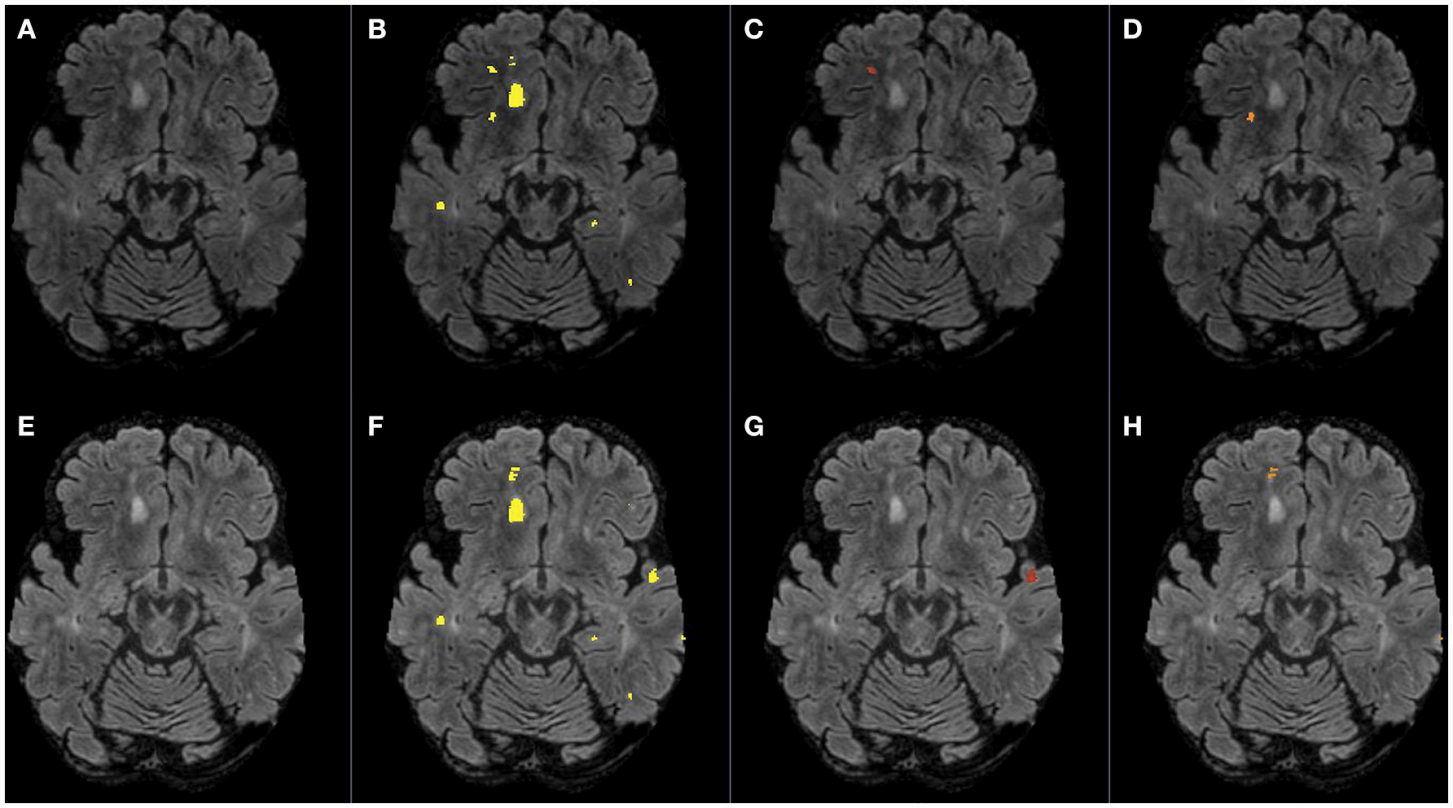
**Bias corrected FLAIR images (A,E)** followed by super-imposed lesion segmentations from: **(B,F)** the expert, **(C)** disappearing lesion, **(D)** shrinking lesion, **(G)** new lesion, and **(H)** enlarging lesion. The first row corresponds to time point 1 and the second row corresponds to time point 2.

Accurate and reliable lesion segmentation based on brain MRI scans is valuable for the diagnosis and monitoring of disease activity in patients with Multiple Sclerosis (MS) (Blystad et al., 2016; Deeks, 2016). The availability of longitudinal MRI data permits an analysis of lesion evolution over time, a potential biomarker of disease progression and treatment efficacy. Figure 1 shows bias corrected FLAIR images of a MS subject scanned twice with an interval of approximately 1 year, along with the expert lesion segmentation followed by the lesion evolution, i.e., the new, disappearing, enlarging, and shrinking lesions. Although expert manual delineation of lesions is considered as the gold standard, it is time consuming and often suffers from intra and inter observer variability (Erbayat Altay et al., 2013). To alleviate this problem, several automatic methods have been proposed in the literature to segment MS lesions. Interestingly, the vast majority of automatic methods are based on a single time point (cross-sectional) and relatively few methods take into account multiple time points (longitudinal) (Llado et al., 2012; Garcia-Lorenzo et al., 2013). Executing a cross-sectional method for each time point would indeed produce the longitudinal measures of interest, but such measures are less reliable as each time point is processed independently. Longitudinal methods incorporate both spatial and temporal information and are expected to be more reliable. Based on the underlying approach, longitudinal methods could be categorized in three different groups: change detection (Gerig et al., 2000; Welti et al., 2001; Prima et al., 2002; Rey et al., 2002; Bosc et al., 2003; Elliott et al., 2013), 4D connectivity (Metcalf et al., 1992; Bernardis et al., 2013) and outlier detection (Solomon and Sood, 2004; Ait-Ali et al., 2005) in multiple time points. Pre-processing of input MR images in these three groups is generally performed and consists of registration to a reference image or a common space, skull stripping, bias field correction and intensity normalization.

Change detection methods primarily aim to detect MS activity by statistical analysis of image features or by measuring local volume variation. Statistical analysis can be performed in an unsupervised or supervised manner. Unsupervised approaches detect significant changes in the intensities between consecutive scans by either analysing the corresponding patches of two time points (Bosc et al., 2003), or performing clustering on the extracted spatial and temporal features from longitudinal images (Gerig et al., 2000; Welti et al., 2001; Prima et al., 2002). The main drawback with unsupervised approaches is that they assume perfect registration and intensity normalization. Supervised approaches learn the desired change from a training dataset; for instance, in Elliott et al. (2013), a random forest discriminative classifier was trained to learn relevant features (intensity, size, and contextual information) related to new lesions and then use these features to segment them. The main drawback with this approach is that it often requires that the training dataset is large enough in order to capture all the distinctive features of the lesions to be segmented. To avoid the need for extracting image features, changes between consecutive images could be directly detected by measuring local volume variations. To this end, a Jacobian operator could be applied to the local deformation field obtained after non-rigid registration between the two time points. Although this approach has proven to be invariant to registration errors, it has given poor results for lesion segmentation (Rey et al., 2002).

Four-dimensional connectivity methods use voxel association in space and time to simultaneously segment and track lesion evolution. For example, Metcalf et al. (1992) segments the lesions in two time points by clustering voxels that are both spatially and temporally adjacent to each other. The main disadvantage of this approach is that it often results in substantial false lesion segmentation. A more advanced method from the same family is based on spectral graph partitioning Bernardis et al. (2013). It constructs a 3D graph in which spatial pairwise affinities characterize lesions and background, and temporal affinities between adjacent time points represent lesion evolution direction. This graph is segmented into lesions and non-lesions via spectral clustering by maximizing the force within-group attraction and between-group repulsion. The drawback of this approach is that it cannot discriminate between consistent artifacts and lesions.

Outlier detection methods are based on the fact that MS lesions are hyper-intense on T2-weighted and fluid-attenuated inversion recovery (FLAIR) brain MRI scans and thus could be detected as an outlier to normal tissue class intensities distribution. For example, a joint expectation-maximization (EM) based approach such as in Ait-Ali et al. (2005) models the healthy brain tissue classes across the time points as a Gaussian mixture model (GMM) using a 4D (3D + time) intensity histogram. The parameters of the model are optimized via a modified version of the EM algorithm referred to as STREAM. After convergence, the lesions are extracted as outliers to healthy tissue classes using Mahalanobis distance and some prior information. In this approach the lesion segmentation is largely dependent on the choice of the Mahalanobis distance parameter and does not target lesion evolution, which is clinically relevant (Ait-Ali et al., 2005). Another approach using outlier detection is based on the hidden Markov model (HMM) technique as in Solomon and Sood (2004). Initially, EM segments the first time point into different tissue classes including lesions, which are then manually corrected. Subsequently, using a lesion growth transition model and outlier detection sensor model, lesions are segmented in the following time points. The transition model enforces consistent lesion segmentation; however, it was validated only on simulations with exponential lesion growth.

In this paper, we present MSmetrix-long: an iterative white matter (WM) lesion segmentation method based on a joint EM framework that takes as input clinically acquired 3D T1-weighted and 3D FLAIR images of two time points. The proposed framework is fully automated, unsupervised and models the lesion evolution as GMM between two time points, thereby simultaneously segmenting new, enlarging, disappearing, shrinking and static lesions. The method is validated for accuracy and reproducibility on two different datasets that are representative for clinically feasible acquisition protocols.

## 2. Methods

**Figure 2:**
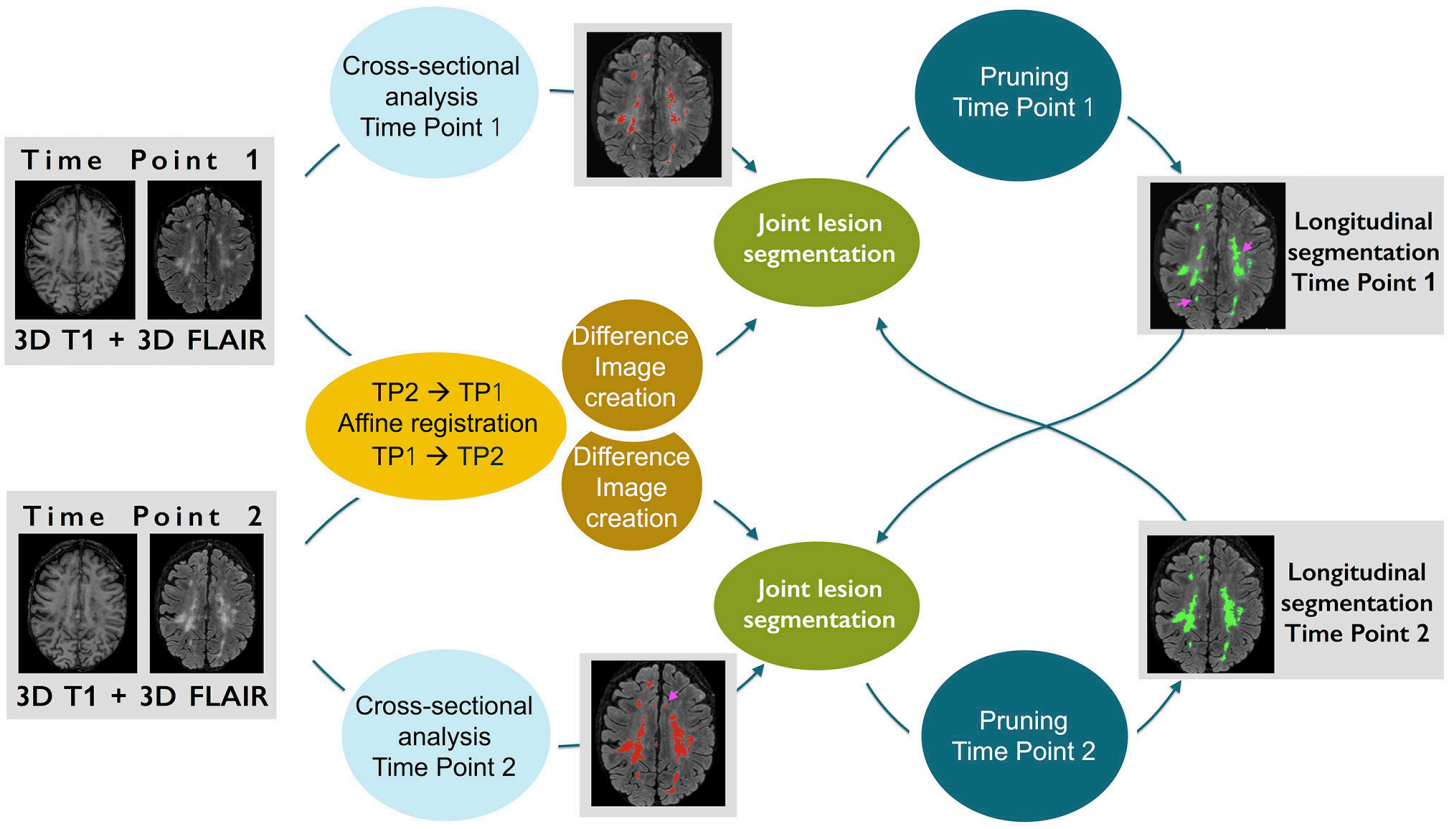
**An illustrative example explaining the different steps of our method**. The pink arrows in the longitudinal lesion segmentation at time point 1 show the recovered lesions using the second time point lesion segmentation and difference image information.

The MSmetrix-long pipeline analyses the MS lesions evolution between two time points based on 3D T1-weighted and 3D FLAIR image acquired at each time point. The pipeline has four steps: (1) Cross-sectional analysis, that segments the individual time points into gray matter (GM), WM, cerebro-spinal fluid (CSF), and lesions, (2) FLAIR based difference image, which is created by subtracting the FLAIR images of both time points after bias correction, co-registration and intensity normalization, (3) Joint lesion segmentation, that aims to improve the individual time point lesion segmentation using the other time point information on tissue and lesion segmentation (initialized using step-1 results) and difference image obtained from step-2, (4) a pruning step, that refines the lesion segmentation obtained in the step-3 to eliminate non-lesions candidates. Figure 2 presents an illustrative explanation of these steps. Steps (3) and (4) are performed sequentially in both directions, by using one time point as reference and then the other. These steps are also iterated, by changing the input lesion segmentation used as prior. Only for the first iteration, the lesion segmentations priors come from the cross-sectional pipeline in step-3, while from the second iteration onwards lesion segmentations from previous iteration are used to initialize the lesion priors for the current iteration. The convergence of our method is decided when the relative lesion segmentation difference between the current and previous iteration is negligible. It takes generally three iterations for the algorithm to converge. The following sections explain the different steps in more detail.

### 2.1. Cross-sectional analysis

Image segmentation is performed independently for each time point using the cross-sectional pipeline referred to as MSmetrix-cross (Jain et al., 2015). The cross-sectional method iteratively segments the T1-weighted image into GM, WM, and CSF, segments the WM lesions on the FLAIR image as an outlier to normal brain using Mahalanobis distance, and performs lesion filling in the T1-weighted image to improve tissue segmentation at next iteration. After convergence, segmentations of WM, GM, CSF and lesions are created. In addition, bias corrected T1-weighted and FLAIR images are also produced. The segmentation tasks of the MSmetrix-cross are optimized using an EM algorithm (Van Leemput et al., 1999) as implemented in NiftySeg (Cardoso, 2012).

### 2.2. FLAIR based difference image

A FLAIR based difference image is created by image co-registration and intensity normalization. Image co-registration is performed using affine registration, which comprises a rigid registration based on the whole T1-weighted image, followed by a skull based affine registration to avoid small scaling differences, and a final whole brain rigid registration (Smeets et al., 2016). The rigid registration and skull based affine registration use an inverse consistent registration algorithm (Modat et al., 2010). Subsequently, the GM, WM, CSF, lesion segmentation and the bias corrected FLAIR images obtained from the cross-sectional analysis are propagated using the final affine transformation. The matched bias corrected FLAIR images are then corrected for differential bias field as described in Lewis and Fox (2004). Subsequently, the differential bias field corrected images are intensity normalized using a cumulative histogram matching technique Castleman (1995) with the image of time point 1 as reference. A FLAIR based difference image is now created in time point 1 space. To avoid bias toward a specific time point, a second difference image is created, using time point 2 space as reference.

### 2.3. Joint lesion segmentation

The joint lesion segmentation model aims at simultaneous tissue class label segmentation of the images from both time points (see the blocks denoted by “Joint lesion segmentation” in Figure 2). The model is optimized using a joint EM algorithm. In this section we present the model formulation, for more details please see Supplementary Material. We now describe the notations, variables and assumptions used, followed by the model definition and its optimization using joint EM.

#### 2.3.1. Notations, variables, and model assumptions

We assume that image 1, image 2 and difference image are co-registered and have the same voxel size. Additionally, image 1 and in image 2 have identical tissue classes. We denote the set of image intensities for image 1 as *I*_1_ and similarly for image 2 as *I*_2_ and for the directional difference image as *D*. *k*^(1)^ and *k*^(2)^ denote tissue class indices for image 1 and image 2, respectively. The tissue class labels in image 1 and in image 2 are denoted by *L*_1_ and *L*_2_ respectively.

We now specify our model assumptions. A Gaussian mixture model is used on the image intensities of each time point where a Gaussian model is used for each tissue class. Let θ_1_ denote the Gaussian mixture model parameters for the intensities of image 1 and *P*(*I*_1_|*L*_1_, θ_1_) denotes the probabilistic model for image 1. Analogously, the probabilistic model for image 2 is denoted by *P*(*I*_2_|*L*_2_, θ_2_).

**Figure 3:**
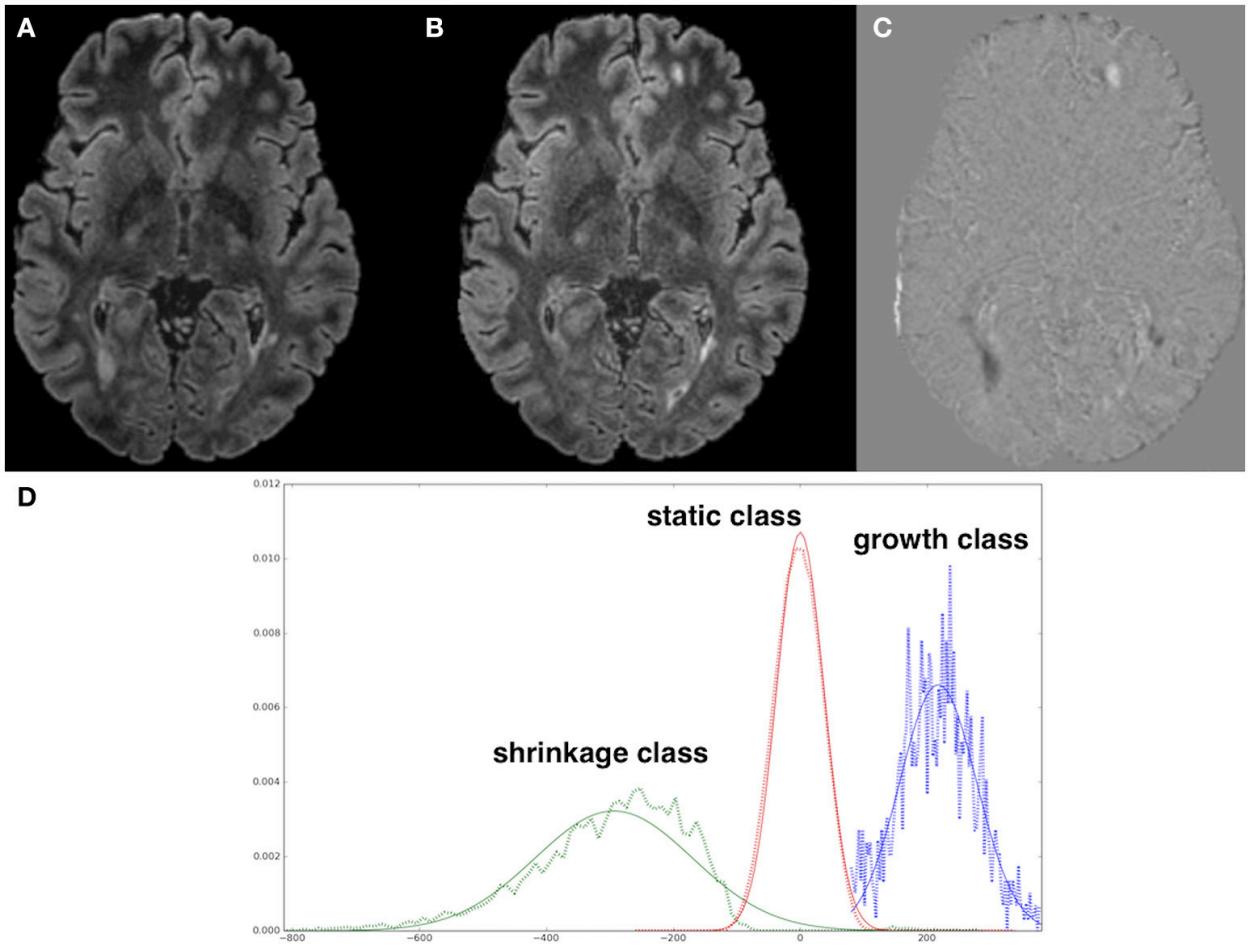
**(A)** Normalized FLAIR image of time point 1, **(B)** FLAIR image of time point 2, **(C)** difference image **(A,B)**, **(D)** histograms of difference image classes with corresponding Gaussian fitting, normalized per class. Note the artifactual difference values at the brain contour (due to subtle differences in brain mask extraction) are excluded by only including WM voxels in the analysis.

We make the underlying assumption that the “difference image” might be independently generated as an image that captures anatomical changes including new lesions or atrophy. The image created by subtracting image 1 from image 2 or vice-versa (after intensity normalization) is one such instance of the difference image. The intensity model of image 1 and image 2 can therefore be reinforced by including a tissue transition model defined on the difference image. As our method focuses on two time point WM lesion segmentation, we only model the transformations between WM and lesions. We assume that the difference image has three different transformations: “static,” “growth,” and “shrinkage.” The static transformation class is defined as a set of voxels in the difference image that are either labeled as WM in both images or lesions. The growth transformation class (describing the new and enlarging lesions) is defined as a set of voxels in the difference image that are labeled as WM in image 1 and lesion in image 2. The shrinkage transformation class (describing the disappearing and shrinking lesions) is defined as a set of voxels in the difference image that are labeled as lesion in image 1 and WM in image 2. For all other possible tissue transformations from image 1 and image 2 a uniform distribution is assumed. Figure 3 shows an illustrative example of the difference image and the histograms of its classes with corresponding Gaussian fitting. Under these assumptions, a Gaussian mixture model for the difference image intensities is used where each transformation class (static, growth, shrinkage) is modeled as Gaussian. The probabilistic model for the difference image is denoted by *P*(*D*|*L*_1_, *L*_2_, ζ), where ζ stands for the Gaussian mixture model parameters for the difference image intensities.

Finally, we assume that we have no prior knowledge on the relationship of the tissue class labels between both images. Therefore, we define the prior probabilities independently for each image. Often these prior probabilities are given by a probabilistic atlas. However, our cross-sectional model provided us with more specific knowledge and hence, we use the probabilistic cross-sectional tissue class segmentations. The prior probabilities on tissue class labels for image 1 and image 2 are denoted by *P*(*L*_1_) and *P*(*L*_2_), respectively.

#### 2.3.2. The model

Under these assumptions, the joint probabilistic model is formulated as follows:

(1) $$\begin{array}{l} P\left(I_{1},I_{2},D,L_{1},L_{2},\gamma \right)\;=\;P\left(I_{1}|L_{1},\theta_{1}\right).\;P\left(I_{2}|L_{2},\theta_{2}\right).\\ \;P\left(D|L_{1},L_{2},\zeta \right).\;P\left(L_{1}\right).\;P\left(L_{2}\right) \end{array}$$

where γ = {θ_1_, θ_2_, ζ}. Our model is optimized by the maximum a posteriori (MAP) problem shown in Equation (2). Since the knowledge of tissue class labels helps in finding the model parameters and vice-versa, we reformulate our MAP problem as presented in Equation (3).

(2) $$\begin{array}{c} {\hat{\gamma }}_{\text{MAP}}=\underset{\gamma }{argmax}\text{ ln }\;P\left(\gamma |I_{1},I_{2},D\right)\;=\;\underset{\gamma }{argmax}\text{ ln}\;\;P\left(I_{1},I_{2},D,\gamma \right) \end{array}$$

(3) $$\begin{array}{c} =\underset{\gamma }{argmax}\text{ln}\underset{L_{1},L_{2}}{\sum_{\text{​}}}P\left(I_{1},I_{2},D,L_{1},L_{2},\gamma \right) \end{array}$$

(4) $$\begin{array}{l} \geq \;\underset{\gamma }{argmax}\underset{L_{1},L_{2}}{\sum_{\text{​}}}P(L_{1},L_{2}|I_{1},I_{2},D,\bar{\gamma }).\\ \text{ ln}\;\;\frac{P\left(I_{1},I_{2},D,L_{1},L_{2},\gamma \right)}{P\left(L_{1},L_{2}|I_{1},I_{2},D,\bar{\gamma }\right)} \end{array}$$

Finally, a lower bound of our model is derived using Jensen's inequality and optimized by the EM algorithm. The Q-function, which is the log likelihood function whose expected value is computed in the E-step can now be written as:

(5) $$\begin{array}{c} Q(\gamma |\bar{\gamma })=E_{L_{1},L_{2}|I_{1},I_{2},D,\bar{\gamma }}\;[\text{ln}\;P\left(I_{1},I_{2},D,L_{1},L_{2},\gamma \right)] \end{array}$$

with the joint posterior distribution $P(L_{1},L_{2}|I_{1},I_{2},D,\bar{\gamma })$. The sum over all possible tissue classes *k*^(2)^ of the joint posterior distribution gives us the soft segmentation of the tissue class at time point 1. Similarly, the sum over all possible tissue classes *k*^(1)^ of the joint posterior distribution gives us the soft segmentation of the tissue class at time point 2.

In the M-step, a new set of values for model parameter γ is computed by maximizing the Q-function (see Supplementary Material for closed form solutions).

### 2.4. Pruning

The soft lesion segmentations obtained from the E-step of the joint EM algorithm are pruned to eliminate non-lesions (such as partial volume effects, artifacts) that share intensities and locations with the potential lesions. Thereto, a priori information on the appearance, location and volume of lesions is incorporated: (1) the lesion intensities should be hyper-intense compared to the WM intensities on bias field corrected FLAIR image, (2) the lesions are in the WM region, and (3) the lesion needs to have a minimum volume of 0.005 ml (empirically determined) to avoid spurious lesion detection. The hyper-intensity is defined as the mean plus two times the standard deviation of WM intensities. The intensities and location of WM region are computed using the WM segmentation from the MSmetrix-cross pipeline. In addition, a priori defined binary mask (defined in the MNI space and consisting of the cerebral cortex and WM in-between the ventricles) is warped to the subject space to remove lesion candidates from these regions that are likely to result in a false lesion segmentation. After the pruning, the soft lesion segmentations are binarized using a threshold of 0.9 (empirically determined) on the posterior probabilities.

### 2.5. Performance tests

#### 2.5.1. Comparison with state-of-the-art methods

We compare MSmetrix-long pipeline with the MSmetrix-cross pipeline to know the gain over the cross-sectional method. Furthermore, we also compare against the longitudinal pipeline of the Lesion Segmentation Toolbox (LST) software package (LST^1^), version 2.0.12, which is implemented in SPM12 (SPM^2^). The longitudinal pipeline of LST, which is referred to as LST-long in this paper, performs individual time point lesion segmentation using the lesion growth algorithm described in Schmidt et al. (2012). The obtained lesion segmentation maps of different time points are coregistered to the baseline scan and are corrected by comparing the relative differences of FLAIR intensities in all lesion maps to produce the final lesion segmentation at each time point (see LST documentation, LST^1^).

For comparison, all three methods were executed on the same datasets and default parameter settings were used. Thus, no parameter tuning was performed at dataset or subject level.

#### 2.5.2. Data

Dataset 1 contains scans from 12 relapsing remitting MS patients on a GE 3T scanner (Discovery MR750), each scanned twice at an interval of approximately 1 year. Therefore, the sample size of dataset 1 equals 24. Each time point contained two a 3D sequences: a CUBE FLAIR (TR: 8000 ms, TE: 165 ms, TI: 2179 ms) and a 3D T1-weighted IR-FSPGR sequence (TR 7.2 ms, TE 450 ms, TI 2.8 ms). Both 3D sequences have voxel resolution close to 1 mm^3^. Expert WM lesion segmentations were created on the baseline FLAIR scan by the experienced neuro-imaging analyst using JIM software tool (JIM^3^), version 6.0. For follow-up scans, baseline lesion segmentation was overlaid on rigidly registered follow-up scan at the beginning, and then the lesion segmentation was adapted according to lesion activities. This study was reviewed and conducted within the guidelines set out in the National Statement on Ethical Conduct in Human Research (2007) in Australia, and approved by University of Sydney Human Research Ethics Committee. All subjects gave written informed consent.

The second dataset, dataset 2 contains scans from 10 MS patients scanned twice, with re-positioning (time interval between two scans is 5~10 min), on each of three different 3T scanners from GE (Discovery MR750w), SIEMENS (Skyra) and PHILIPS (Achieva). Therefore, the sample size of dataset 2 equals 60. The protocol contained two 3D sequences: T1-weighted and FLAIR, and their details are described in Table 1. For this dataset, no expert segmentations were available. This study was carried out in accordance with the recommendations of the “International Conference on Harmonization of Good Clinical Practice (ICH-GCP),” and the applicable Belgian and Dutch legislation. The study was approved by the UZ Brussels ethical committee. All subjects gave written informed consent in accordance with the Declaration of Helsinki.

**Table 1:** **Dataset-2 sequences description for all three scanners**.

**Sequence**	**TR (ms)**	**TE (ms)**	**TI (ms)**	**FOV (mm^2^)**	**Voxel size (mm^3^)**	**No. of slices (sagittal)**
**GE**						
3D T1-weighted FSPGR	7.32	3.14	NA	220 × 220	0.43 × 0.43 × 0.50	328
Fat saturated 3D FLAIR	9500	135.78	2428	240 × 240	0.47 × 0.47 × 0.70	232
**SIEMENS**						
3D T1-weighted MPRAGE	2300	2.29	NA	240 × 240	0.94 × 0.94 × 0.94	176
Fat saturated 3D FLAIR	5000	387	1800	230 × 230	0.45 × 0.45 × 0.90	192
**PHILIPS**						
3D T1-weighted FSPGR	4.93	2.3	NA	230 × 230	0.53 × 0.53 × 0.50	310
Fat saturated 3D FLAIR	4800	276	1650	240 × 240	1.04 × 1.04 × 0.56	321

*NA, Not available*.

#### 2.5.3. Accuracy and reproducibility assessment

The agreement between the expert segmentation and automatic methods on dataset 1 is evaluated at three levels: voxel-by-voxel, lesion-wise and volumetric. Voxel-by-voxel metric includes the Dice similarity index which is defined as the ratio of total number of lesion voxels where both the expert reference and the automatic segmentation agree (true positives) to the mean number of voxels labeled as lesion by the two methods. The lesion-wise metrics include lesion-wise true positive rate (LTPR), false positive rate, F1 score, absolute lesion change difference and Pearson correlation coefficient (PCC). LTPR is defined as the ratio of the total number of lesions where the expert reference and the automatic segmentation intersect to the total number of lesions in the expert reference segmentation. Lesion-wise false positive rate (LFPR) is defined as the ratio of the total number of lesions that are present only in the automatic segmentation to the total number of lesions in the automatic segmentation. Lesion-wise F1 score is defined as the harmonic mean of LTPR and LFPR. Absolute lesion-wise change difference is defined as the absolute difference between the overall lesion-wise change (number of new lesions minus number of disappearing lesions) in the expert lesion segmentation and the automatic segmentation. In this paper, we consider new, disappearing, enlarging and shirking lesions that have size more than 20 voxels and at least one slice which encompasses the lesion presents a minimum of 5 lesion voxels.

Volumetric metrics measure the total lesion volume agreement and consist of the PCC and the absolute volume difference. The absolute volume difference is computed as the absolute difference between the total volume reported by the expert reference segmentation and the corresponding value derived from the automatic method.

The reproducibility of the method is evaluated on dataset 2 by the Dice similarity index of the lesion segmentations at both times points. Moreover, the estimated number of new lesions and the absolute total lesion volume difference is also calculated between time points, which are both expected to be zero in this test-retest scenario.

To determine if there is a statistical difference between MSmetrix-long and LST-long and between MSmetrix-cross and MSmetrix-long methods' performance, two tailed paired Wilcoxon signed-rank test is performed.

## 3. Results

### 3.1. Accuracy results on dataset 1

**Figure 4:**
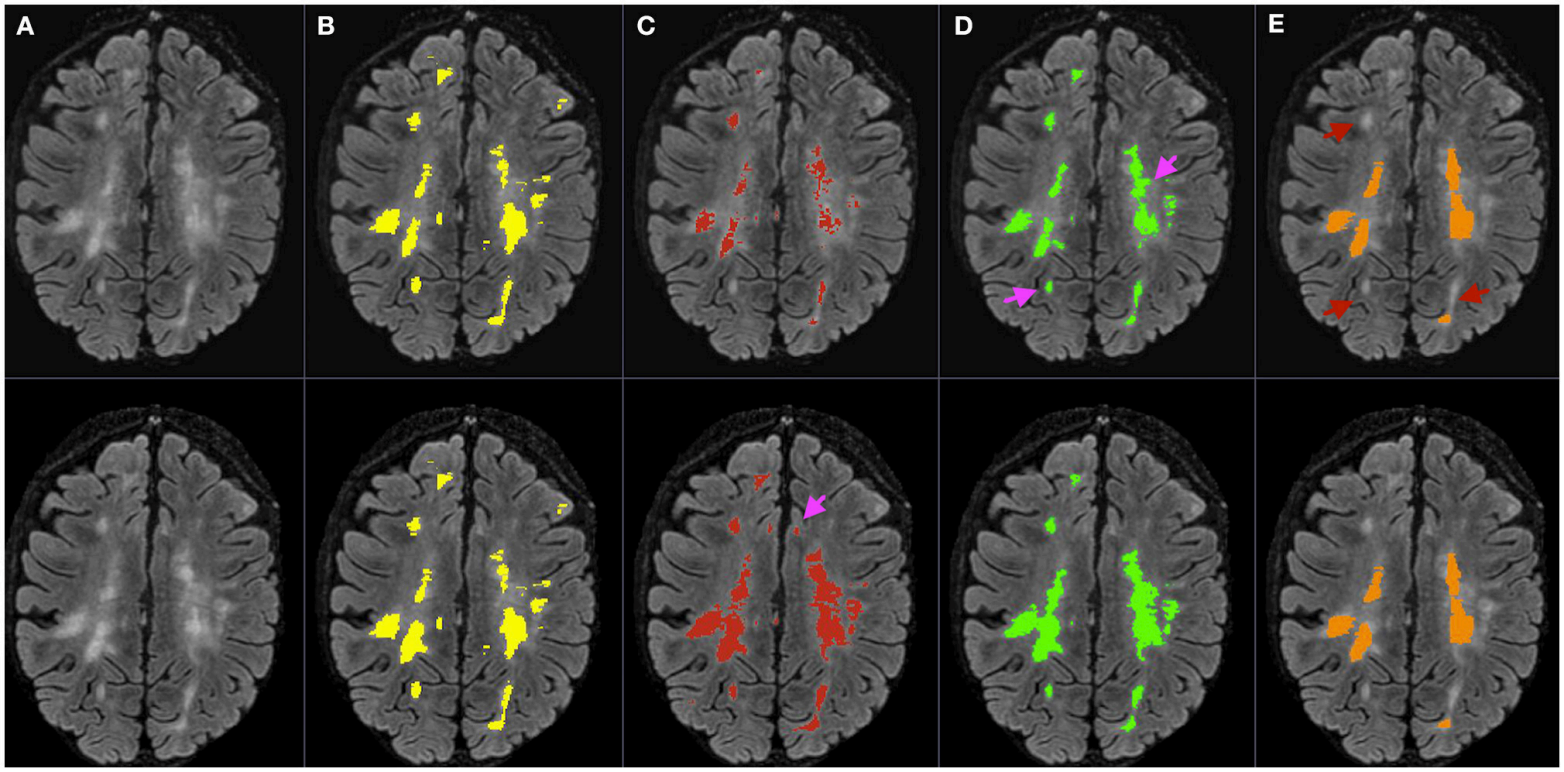
**Bias corrected FLAIR image (A)** followed by super-imposed lesion segmentation from: **(B)** expert segmentation, **(C)** MSmetrix-cross (version 1.4), **(D)** MSmetrix-long, and **(E)** LST-long. The first row corresponds to the lesion segmentation of time point 1 and the second row corresponds to the lesion segmentation of time point 2. Pink arrows specify places where MSmetrix-long has improved in accuracy over MSmetrix-cross and red arrows indicate regions where LST-long has missed lesions.

Figure 4 shows a representative example of lesion segmentation obtained by MSmetrix-cross, MSmetrix-long and LST-long on a patient from dataset 1. By comparing against expert delineations, it can be observed that MSmetrix-long has improved in accuracy over MSmetrix-cross and that LST-long has missed lesions.

**Figure 5:**
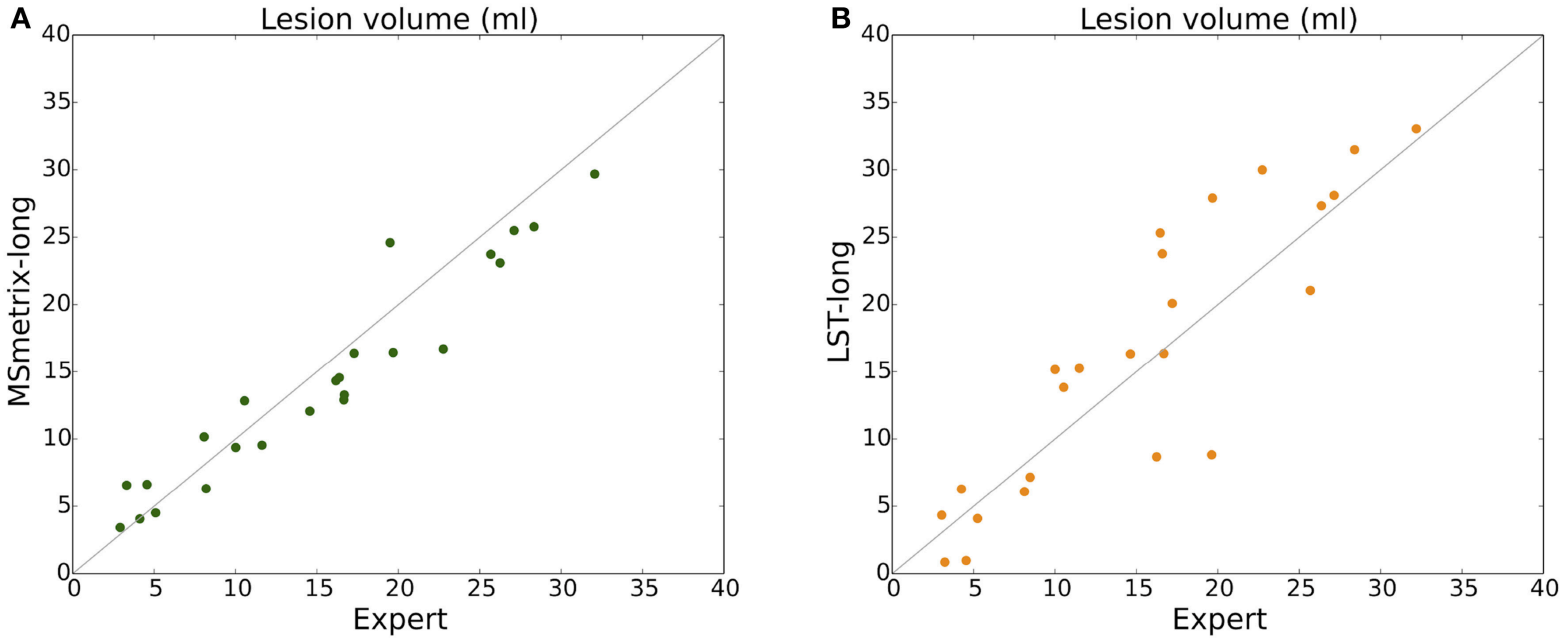
**Scatter plot of total lesion volume (ml) for reference expert segmentation vs. (A)** MSmetrix-long and **(B)** LST-long.

The volumetric correlation of MSmetrix-long and LST-long to the expert reference segmentation can be visualized in Figure 5. MSmetrix-long has a better correlation (PCC = 0.96) with expert reference segmentation compared to LST-long (PCC = 0.88).

Table 2 summarizes the cross-sectional lesion segmentation performance of MSmetrix-cross, MSmetrix-long and LST-long on dataset 1 (*n* = 24) in a quantitative way. MSmetrix-long has improved over MSmetrix-cross in the median Dice, F1 score and LFPR. Compared to LST-long, MSmetrix-long has a higher median Dice, F1 score, LTPR, and PCC, together with lower LFPR and absolute lesion volume difference.

**Table 2:** **Quantitative metrics (voxel-by-voxel, lesion and volumetric level) for measuring the cross-sectional accuracy of the automatic methods MSmetrix-long, MSmetrix-cross and LST-long with respect to expert segmentations on dataset 1 (***n*** = 24)**.

**Automatic method**	**Dice**	**F1 score**	**LTPR**	**LFPR**	**Absolute volume difference (ml)**	**PCC**
MSmetrix-long	0.63 (0.49–0.68)	0.61 (0.54–0.63)	0.50 (0.43–0.59)	0.25 (0.20–0.37)	2.09 (1.77–3.18)	0.96
MSmetrix-cross	0.60 (0.46–0.66)^**^	0.56 (0.52–0.61)^*^	0.57 (0.52–0.65)^**^	0.48 (0.36–0.55)^**^	1.48 (0.81–2.59)	0.95
LST-long	0.60 (0.47–0.65)^*^	0.48 (0.37–0.53)^**^	0.42 (0.30–0.52)^*^	0.40 (0.30–0.47)	2.66 (1.52–4.84)^*^	0.88

*Except PCC, all metrics are reported in median (first quartile–third quartile). LTPR, lesion-wise true positive rate; LFPR, lesion-wise false positive rate; PCC, Pearson correlation coefficient*.

* *Values significantly different from MSmetrix-long (paired Wilcoxon signed-rank test with p < 0.05 significance level)*.

** *Values significantly different from MSmetrix-long (paired Wilcoxon signed-rank test with p < 0.01 significance level)*.

Table 3 summarizes the lesion-wise change accuracy performance of MSmetrix-cross, MSmetrix-long and LST-long on dataset 1 in a quantitative way. In case of new lesions, MSmetrix-long has improved over MSmetrix-cross in the median F1 score and LFPR. Compared to LST-long, MSmetrix-long has a higher median F1 score and LTPR. In case of enlarging lesions, MSmetrix-long has improved over MSmetrix-cross in the median LFPR, with marginally better F1 score. Compared to LST-long, MSmetrix-long has a higher median F1 score, LTPR, and LFPR. When new and enlarging lesions are combined, MSmetrix-long has better correlation (PCC = 0.77) with the expert segmentations compared to MSmetrix-cross (PCC = 0.63) and LST-long (PCC = 0.53). In case of absolute lesion-wise change difference, MSmetrix-long has marginally better performance over MSmetrix-cross and LST-long, however, with better correlation with the lesion-wise change difference of the expert segmentations (PCC = 0.84) compared to MSmetrix-cross (0.65) and LST-long (0.72).

**Table 3:** **Lesion-wise quantitative metrics for measuring the lesion change accuracy of the automatic methods MSmetrix-long, MSmetrix-cross and LST-long with respect to expert lesion segmentations changes on dataset 1**.

	**New lesions**			**Enlarging lesions**			**New and enlarging lesions**
	**F1 score**	**LTPR**	**LFPR**	**F1 score**	**LTPR**	**LFPR**	**PCC**
MSmetrix-long	0.42 (0-0.55)	0.33 (0–0.60)	0 (0–0.38)	0.69 (0.56–0.81)	0.62 (0.53–0.69)	0.16 (0–0.51)	0.77
MSmetrix-cross	0.20 (0.0–0.62)	0.33 (0–0.52)	0.50 (0.31–0.75)	0.68 (0.58–0.80)	0.59 (0.53–0.69)	0.24 (0.15–0.43)	0.63
LST-long	0 (0-0.43)	0 (0–0.29)	0 (0–0)	0.60 (0.51–0.69)	0.50 (0.35–0.60)	0.33 (0.15–0.51)	0.53
**Absolute lesion-wise change difference**							
		**PCC**					
MSmetrix-long	1 (1-3.5)	0.84					
MSmetrix-cross	1.5 (1–3.75)	0.65					
LST-long	2 (1-3.5)	0.72					

*Except PCC, all metrics are reported in median (first quartile–third quartile). PCC, Pearson correlation coefficient. Here, the t-test is not performed, as the sample size is small (n = 12)*.

### 3.2. Reproducibility results on dataset 2

**Figure 6:**
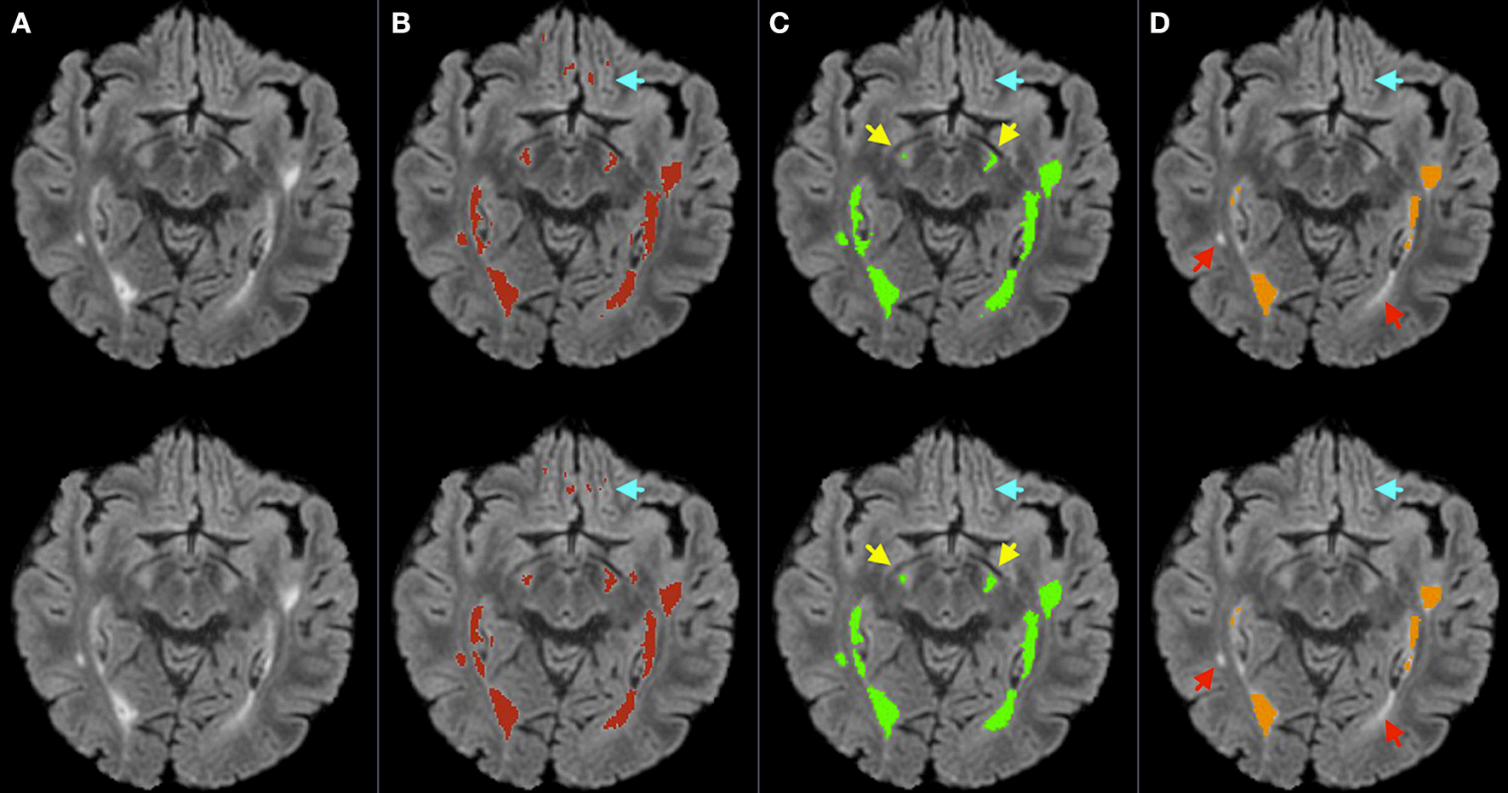
**Bias corrected FLAIR image (A)** followed by super-imposed lesion segmentation from: **(B)** MSmetrix-cross (version 1.4), **(C)** MSmetrix-long, and **(D)** LST-long. The first row corresponds to the lesion segmentation of time point 1 and the second row corresponds to the lesion segmentation of time point 2. Cyan arrows show some false positives in MSmetrix-cross, which are absent in MSmetrix-long. Yellow arrows specify places where MSmetrix-long has consistently segmented some small lesions and red arrows indicate regions where LST-long misses some potential lesions.

Figure 6 shows an example of lesion segmentation obtained by MSmetrix-cross, MSmetrix-long and LST-long on a patient from dataset 2 (*n* = 60). Both MSmetrix-long and LST-long are more consistent in lesion segmentation compared to MSmetrix-cross. Compared to LST-long, MSmetrix-long also shows better reproducibility in segmenting small lesions. Quantitatively, LST-long has the best median Dice with zero error in detecting new lesions and absolute volume difference between both time points. MSmetrix-long has improved in the median Dice, with median error in detecting new lesions and absolute volume difference over MSmetrix-cross. The reproducibility of LST-long is highest because it segments the most certain hyper-intense lesions in both time points at the expense of missing substantial amount of less hyper-intense lesions as shown in Figure 6.

## 4. Discussion and conclusions

Accurate and consistent lesion segmentation is very important in monitoring the MS disease progression. As manual lesion segmentation is time consuming and suffers from inter- and intra-rater variability, automated methods have the advantage of being fast and consistent. The vast majority of automatic methods are cross-sectional in nature and the average accuracy (Dice) of these methods is sufficiently high, however, these cross-sectional methods seldom report results on the lesion evolution accuracy and this hinders a fair comparison of our method against them. Moreover, another factor to consider is whether the segmentation method is supervised or unsupervised. We compare our unsupervised method with other unsupervised methods only because supervised methods often require a representative training dataset, including expert segmentation, in order to build a model that can be used on new patients for lesion segmentation. This training dataset is very difficult to build because MS lesions have all possible shapes, intensities and are heterogeneously distributed in the WM. Moreover, the new image to be segmented should be well represented in the training dataset which is not always possible. Two well-known publicly available unsupervised MS lesion segmentation tools are Lesion-TOADS (Shiee et al., 2010) and LST. We choose LST because of two reasons: (1) in a previous paper (Jain et al., 2015), we have shown that our cross-sectional method (MSmetrix-cross) had a better performance compared to Lesion-TOADS in terms of accuracy and reproducibility. Since in this paper we also report results from our cross-sectional method, we decided that the comparison with Lesion-TOADS is not required, (2) only LST tool has a longitudinal MS lesion segmentation pipeline. Thus it is logical to compare MSmetrix-long with LST-long as both methods are unsupervised and longitudinal in nature.

In this paper, MSmetrix-long pipeline combines both spatial and temporal relationships of lesions for accurate and consistent lesion segmentation. The spatial relationship is based on Markov Random Field and is incorporated in MSmetrix-cross. The temporal relationship is modeled in a joint lesion segmentation, which uses difference image and cross-sectional lesion segmentations of two time points. The difference image models the growth and shrinkage of lesions and thus helps in recovering those lesions that are missed by the cross-sectional lesion segmentation. In addition, if a lesion is present in both time points but has been segmented in only one of the time point, then the joint lesion segmentation facilitates the recovery of that lesion at the other time point. Moreover, brain atrophy has also minimal impact on the performance of MSmetrix-long because (1) atrophy is generally small and global in nature (2) it occurs near the CSF boundary and these transitions *i.e*. (CSF → GM and CSF → WM) are excluded in the difference image GMM model, (3) we tested global non-rigid registration in addition to affine registration, i.e., non-rigid registration only on a coarse level, to accommodate for the atrophy and we found out that it has a minimal, but potentially negative impact on the final lesion segmentation. Therefore, to gain computational efficiency we excluded this global non-rigid registration from MSmetrix-long pipeline. Furthermore, if the subject has been scanned more than twice, MSmetrix-long can easily handle this by processing consecutive time points in pairs.

Among the methods proposed in the literature for longitudinal lesion segmentation, our approach has some similarities to Elliott et al. (2013) and Ait-Ali et al. (2005), which are also based on EM frameworks. In contrast with Elliott et al. (2013), our method is unsupervised and can segment new, enlarging, disappearing and shrinking lesions. As opposed to Ait-Ali et al. (2005), our joint EM model takes cross-sectional lesion segmentation as prior information on the lesion class in both time points and processes each time point in its own space to avoid bias in the lesion segmentation.

In order to evaluate the effect of the pruning step, we also calculated the cross-sectional accuracy (Dice, LTPR and LFPR) of MSmetrix-long after the joint lesion segmentation step. The Dice, LTPR, and LFPR (reported in median (first quartile–third quartile)) after the joint lesion segmentation step are 0.60 (0.45–0.65), 0.64 (0.54–0.69), and 0.81 (0.72–0.87) respectively. Comparing these results with the the voxel-by-voxel accuracy of MSmetrix-long after the pruning step (see Table 2), we observe that the pruning step increases the overall Dice score by decreasing the false positive rate at the expense of a decrease in true positive rate.

In order to investigate the cause of low LTPR for cross-sectional accuracy of MSmetrix-long compared to MSmetrix-cross (see Table 2), we calculated the average LTPR for small (0.003–0.01 ml), medium (0.01–0.05 ml) and large (>0.05 ml) lesion volumes. The average LTPR for MSmetrix-long and MSmetrix-cross for small lesions is 0.13 and 0.27 respectively, followed by medium lesions 0.30 and 0.37 and large lesions 0.75 and 0.81. It can be seen that MSmetrix-long misses more small and medium size lesions. The primary cause of missing these lesions is that they are either iso-intense with GM intensities (thus missed by intensity threshold mask used in the pruning step) or they are removed by the binary false positive mask (used in the pruning step). However, it is important to note that both intensity threshold mask and binary false positive mask play a key role in reducing the false positives as described in the previous paragraph.

One important aspect of MSmetrix-long is that its performance is dependent on the cross-sectional lesion segmentation. This suggests that if MSmetrix-cross has either consistently missed a lesion, or segmented a non-lesion at both time points, then it will be either missed or retained by MSmetrix-long, respectively. As presented in the result section, MSmetrix-long is more accurate and reproducible than MSmetrix-cross. The increase in cross-sectional accuracy (Dice, F1 score) and lesion change accuracy for new lesions (F1 score) is due to the reduction in LFPR using the lesion segmentation information from the other time point. For enlarging lesions, a marginal increase in the median F1 score is observed for MSmetrix-long due to larger differences in the lesion segmentation boundary between the expert and MSmetrix-long. MSmetrix-long has also slightly better absolute lesion-wise change difference compared to MSmetrix-cross primarily due to a reduction in LFPR. A modest decrease in the absolute volume difference is due to the under-segmentation of lesions by MSmetrix-long (Figure 5) and the elimination of a few lesions that are close to the cerebral cortex. Interestingly, a substantial LFPR in MSmetrix-cross suggests that the false lesions compensate toward missed lesions volume resulting in a lower absolute volume difference compared to MSmetrix-long. The significant improvement in reproducibility (Dice, number of new lesions and absolute volume difference) of MSmetrix-long could also be explained by the benefit of using the lesion segmentation of the other time point.

In comparison to LST-long, MSmetrix-long is more accurate (Dice, F1 score) and slightly less reproducible. Cross-sectionally, LST-long has higher absolute volume difference and LFPR; lower LTPR and F1 score on dataset 1. The high absolute volume difference of LST-long could be explained by the over-segmentation of lesion boundaries. A high lesion-wise false positive rate of LST-long could be explained by the segmentation of FLAIR artifacts or cortical foldings as lesions. For the lesion change accuracy, MSmetrix-long has superior performance for all measures compared to LST-long. This could be explained by the fact that LST-long segments the most hyper-intense lesions and is thus very consistent (see Table 4), but misses many small less hyper-intense lesions (Figures 4, 6).

**Table 4:** **The Dice score, the number (Nr.) of new lesions and the absolute volume difference (Abs. vol. diff.) between both time points for measuring the accuracy of the automatic methods MSmetrix-long, MSmetrix-cross and LST-long on dataset 2**.

	**Dice**	**Nr. of new les**	**Abs. vol. diff. (ml)**
MSmetrix-long	0.89 (0.85–0.91)	0 (0–1)	0.11 (0.03–0.32)
MSmetrix-cross	0.69 (0.56–0.73)^**^	3.5 (1–5)^**^	0.3 (0.17–0.54)^*^
LST-long	1 (1–1)^**^	0 (0–0)^**^	0 (0–0.01)^**^

*All metrics are reported as median (first quartile–third quartile)*.

* *Values significantly different from MSmetrix-long (paired Wilcoxon signed-rank test with p < 0.05 significance level)*.

** *Values significantly different from MSmetrix-long (paired Wilcoxon signed-rank test with p < 0.01 significance level)*.

In conclusion, we have presented MSmetrix-long: an iterative two time point WM lesion segmentation method based on a joint EM framework using two time points. The proposed method is unsupervised and can segment new, enlarging, disappearing, shrinking and static lesions. We first analyse both time points separately followed by a joint lesion segmentation, which models the lesion evolution as a Gaussian mixture model. The accuracy and reproducibility of MSmetrix-long is compared with MSmetrix-cross and the publicly available lesion segmentation tool LST-long on two datasets that are representative for clinically feasible acquisition protocols. MSmetrix-long has outperformed MSmetrix-cross. Compared to LST-long, MSmetrix-long has better accuracy and similar reproducibility.

## Author contributions

SJ, AR, DMS, SV, FM, and DS contributed to the design and analysis of the work; MC, JD, CW, and MB contributed to the data acquisition; SJ, AR and DMS wrote the paper; all authors revised the manuscript critically for important intellectual content.

## Funding

This study has been supported by TRANSACT (FP7-PEOPLE-2012-ITN-316679), CENTER-TBI (FP7-COOPERATION-2013-602150) and BRAINPATH (FP7-PEOPLE-2013-IAPP-612360).

### Conflict of interest statement

The authors declare that the research was conducted in the absence of any commercial or financial relationships that could be construed as a potential conflict of interest.

## Footnotes

^1^www.statistical-modelling.de/lst.html

^2^http://www.fil.ion.ucl.ac.uk/spm/software/spm12

^3^http://www.xinapse.com

## References

[B1] Aït-Ali L. S. Prima S. Hellier P. Carsin B. Edan G. Barillot C. (2005). STREM: a robust multidimensional parametric method to segment MS lesion in MRI. Med. Image Comput. Comput. Assist. Interv. 8, 409–416. 10.1007/11566465_51 16685872

[B2] Bernardis E. Pohl K. M. Davatzikos C. (2013). Extracting evolving pathologies via spectral clustering, in Information Processing in Medical Imaging, 23rd International Conference, IPMI (Asilomar), 680–691. 10.1007/978-3-642-38868-2_57 PMC11073859 24684009

[B3] Blystad I. Håkansson I. Tisell A. Ernerudh J. Smedby Ö. Lundberg P.. (2016). Quantitative MRI for analysis of active multiple sclerosis lesions without gadolinium-based contrast agent. Am. J. Neuroradiol. 37, 94–100. 10.3174/ajnr.A4501 26471751 PMC7960216

[B4] Bosc M. Heitz F. Armspach J. P. Namer I. Gounot D. Rumbach L. (2003). Automatic change detection in multimodal serial MRI: application to multiple sclerosis lesion evolution. Neuroimage 20, 643–656. 10.1016/S1053-8119(03)00406-3 14568441

[B5] Cardoso M. J. (2012). NiftySeg: Statistical Segmentation and Label Fusion Software Package. Available online at: http://niftyseg.sourceforge.net/index.html [Accessed: 25-04-2016].

[B6] Castleman K. R. (1995). Digital Image Processing, 2nd Edn. Englewood: Prentice Hall.

[B7] Deeks E. D. (2016). Dimethyl fumarate: a review in relapsing-remitting MS. Drugs 76, 243–254. 10.1007/s40265-015-0528-1 26689201

[B8] Elliott C. Arnold D. L. Collins D. L. Arbel T. (2013). Temporally consistent probabilistic detection of new multiple sclerosis lesions in brain MRI. IEEE Trans. Med. Imaging 32, 1490–1503. 10.1109/TMI.2013.2258403 23613032

[B9] Erbayat Altay E. Fisher E. Jones S. E. Hara-Cleaver C. Lee J. C. Rudick R. A. (2013). Reliability of classifying multiple sclerosis disease activity using magnetic resonance imaging in a multiple sclerosis clinic. JAMA Neurol. 70, 338–344. 10.1001/2013.jamaneurol.211 23599930 PMC3792494

[B10] García-Lorenzo D. Francis S. Narayanan S. Arnold D. L. Collins D. L. (2013). Review of automatic segmentation methods of multiple sclerosis white matter lesions on conventional magnetic resonance imaging. Med. Image Anal. 17, 1–18. 10.1016/j.media.2012.09.004 23084503

[B11] Gerig G. Welti D. Guttmann C. R. G. Colchester A. C. F. Székely G. (2000). Exploring the discrimination power of the time domain for segmentation and characterization of active lesions in serial MR data. Med. Image Anal. 4, 31–42. 10.1016/S1361-8415(00)00005-0 10972319

[B12] Jain S. Sima D. M. Ribbens A. Cambron M. Maertens A. Van Hecke W.. (2015). Automatic segmentation and volumetry of multiple sclerosis brain lesions from MR images. Neuroimage Clin. 8, 367–375. 10.1016/j.nicl.2015.05.003 26106562 PMC4474324

[B13] Lewis E. B. Fox N. C. (2004). Correction of differential intensity inhomogeneity in longitudinal MR images. Neuroimage 23, 75–83. 10.1016/j.neuroimage.2004.04.030 15325354

[B14] Lladé X. Ganiler O. Oliver A. Martí R. Freixenet J. Valls L.. (2012). Automated detection of multiple sclerosis lesions in serial brain MRI. Neuroradiology 54, 787–807. 10.1007/s00234-011-0992-6 22179659

[B15] Metcalf D. Kikinis R. Guttmann C. Vaina L. Jolesz F. (1992). 4D connected component labelling applied to quantitative analysis of MS lesion temporal development, in Annual International Conference of the IEEE Engineering in Medicine and Biology Society (Paris), 945–946.

[B16] Modat M. Ridgway G. R. Taylor Z. A. Lehmann M. Barnes J. Hawkes D. J.. (2010). Fast free-form deformation using graphics processing units. Comput. Methods Prog. Biomed. 98, 278–284. 10.1016/j.cmpb.2009.09.002 19818524

[B17] Prima S. Ayache N. Janke A. Francis S. J. Arnold D. L. Collins D. L. (2002). Statistical analysis of longitudinal MRI data: applications for detection of disease activity in MS. Med. Image Comput. Comput. Assist. Interv. 2488, 363–371. 10.1007/3-540-45786-0_45

[B18] Rey D. Subsol G. Delingette H. Ayache N. (2002). Automatic detection and segmentation of evolving processes in 3D medical images: application to multiple sclerosis. Med. Image Anal. 6, 163–179. 10.1016/S1361-8415(02)00056-7 12045002

[B19] Schmidt P. Gaser C. Arsic M. Buck D. Förschler A. Berthele A.. (2012). An automated tool for detection of FLAIR-hyperintense white-matter lesions in Multiple Sclerosis. Neuroimage 59, 3774–3783. 10.1016/j.neuroimage.2011.11.032 22119648

[B20] Shiee N. Bazin P. L. Ozturk A. Reich D. S. Calabresi P. A. Pham D. L.. (2010). A topology-preserving approach to the segmentation of brain images with multiple sclerosis lesions. Neuroimage 49, 1524–1535. 10.1016/j.neuroimage.2009.09.005 19766196 PMC2806481

[B21] Smeets D. Ribbens A. Sima D. M. Cambron M. Horakova D. Jain S.. (2016). Reliable measurements of brain atrophy in individual patients with multiple sclerosis. Brain Behav. 6:e00518. 10.1002/brb3.518 27688944 PMC5036437

[B22] Solomon J. Sood A. (2004). 4-D lesion detection using expectation-maximization and hidden markov model, in IEEE International Symposium Biomedical Imaging (Arlington, VA), 125–128.

[B23] Van Leemput K. Maes F. Vandermeulen D. Suetens P. (1999). Automated model-based tissue classification of mr images of the brain. IEEE Transactions of the Medical Imaging 18, 897–908. 10.1109/42.811270 10628949

[B24] Welti D. Gerig G. Radü E.-W. Kappos L. Székely G. (2001). Spatio-temporal segmentation of active multiple sclerosis lesions in serial MRI data, in Information Processing in Medical Imaging, 17th International Conference, IPMI (Davis, CA), 438–445.

