# Supplementary material for: Two Time Point MS Lesion Segmentation in Brain MRI: An Expectation-Maximization Framework
Source: Front Neurosci. 2016 Dec 19;10:576. doi: 10.3389/fnins.2016.00576 (PMC5165245; doi:10.3389/fnins.2016.00576)
Supplement: Supplementary file 1 [file Presentation1.pdf]

Supplementary Material: Two Time Point MS Lesion Segmentation in Brain MRI: An Expectation-Maximization Framework

Saurabh Jain*, Annemie Ribbens, Diana M. Sima, Melissa Cambron, Jacques De Keyser, Chenyu Wang, Michael H. Barnett, Sabine Van Huffel, Frederik Maes, Dirk Smeets

*Correspondence:
Author Name: Saurabh Jain
saurabh.jain@icometrix.com

1 DETAILS OF THE JOINT LESION SEGMENTATION MODEL AND ALGORITHM

1.1 Notations, variables and model assumptions

We assume that image 1, image 2 and difference image have the number of voxels N_J. Let $I_1 = \{y_j^{(1)}$ | j $\in \{1, .., N_J\}\}$, $I_2 = \{y_j^{(2)}$ | j $\in \{1, .., N_J\}\}$ and $D = \{y_j^{(D)}$ | j $\in \{1, .., N_J\}\}$ be the set of image intensities with $y_j^{(1)}$, $y_j^{(2)}$ and $y^{(D)}$ being the intensities corresponding to voxel j in image 1, image 2, and difference image respectively.

We assume that total number of tissue classes in both images is denoted by N_K. Let $k = \{1, .., N_K\}$ be the set of tissue classes with $k^{(1)}$ and $k^{(2)}$ denoting a tissue class index for image 1 and image 2, respectively such that $k^{(1)}$ and $k^{(2)} \in \{1, .., N_K\}$. The tissue class label in image 1 is defined as: $L_1 = \{l_{j,k^{(1)}}^{(1)}$ | j$\in\{1, .., N_J\}$, $k^{(1)} \in \{1, .., N_K\}\}$; $l_{j,k^{(1)}}^{(1)}$ is 1 if voxel j has tissue class label $k^{(1)}$ in I_1, 0 otherwise; $l_{j,k^{(1)}}^{(1)}$ is subjected to the constraint: $\sum_{k^{(1)}} l_{j,k^{(1)}}^{(1)} = 1$. Similarly, the set of tissue class labels in image 2 are denoted by L_2 and defined in the same way as for L_1.

Let $\{\mu_{k^{(1)}}^{(1)}, \sigma_{k^{(1)}}^{2(1)}\}$ be the mean and variance of the Gaussian model parameters for tissue class $k^{(1)}$ and let θ_1 denote the Gaussian mixture model parameters for the intensities of image 1. The Gaussian mixture model for the image 1 intensities can now be defined as:

$$P(I_1|L_1, \theta_1) = \prod_{j,k^{(1)}} \mathcal{N}\left(y_j^{(1)}|\mu_{k^{(1)}}^{(1)}, \sigma_{k^{(1)}}^{2(1)}\right)^{l_{j,k^{(1)}}^{(1)}} \quad \text{(S1)}$$

Analogously, the Gaussian mixture model for the image 2 intensities is defined as:

$$P(I_2|L_2, \theta_2) = \prod_{j,k^{(2)}} \mathcal{N}\left(y_j^{(2)}|\mu_{k^{(2)}}^{(2)}, \sigma_{k^{(2)}}^{2(2)}\right)^{l_{j,k^{(2)}}^{(2)}} \quad \text{(S2)}$$

The Gaussian mixture model for the difference image intensities is defined as:

$$P(D|L_1, L_2, \zeta) = \begin{cases} \prod\limits_{j,k^{(1)},k^{(2)}} \mathcal{N}\left(y_j^{(D)}|\mu^{(D)}_{k^{(1)},k^{(2)}}, \sigma^{2(D)}_{k^{(1)},k^{(2)}}\right)^{l^{(1)}_{j,k^{(1)}},\, l^{(2)}_{j,k^{(2)}}} & \text{, with } k^{(1)} \text{ and } k^{(2)} \in \{\text{WM, lesion}\} \\ \propto\ 1 & \text{, } \forall \text{ other } k^{(1)} \text{ and } k^{(2)} \text{ combinations.} \end{cases} \quad \text{(S3)}$$

and the parameter $\zeta = \{\theta_{static}, \theta_{growth}, \theta_{shrinkage}\}$ has

$$\theta_{static} = \{\mu^{(D)}_{static}, \sigma^{2(D)}_{static}\}, \qquad \text{if} \begin{cases} k^{(1)} = k^{(2)} = \text{WM or} \\ k^{(1)} = k^{(2)} = \text{lesion} \end{cases} \quad \text{(S4)}$$

$$\theta_{growth} = \{\mu^{(D)}_{growth}, \sigma^{2(D)}_{growth}\}, \qquad \text{if } k^{(1)} = \text{WM, } k^{(2)} = \text{lesion} \quad \text{(S5)}$$

$$\theta_{shrinkage} = \{\mu^{(D)}_{shrinkage}, \sigma^{2(D)}_{shrinkage}\}, \quad \text{if } k^{(1)} = \text{lesion, } k^{(2)} = \text{WM} \quad \text{(S6)}$$

The prior probabilities on tissue class labels for the image 1 and image 2 are denoted by $P(L_1)$ and $P(L_2)$, and are defined as:

$$P(L_1) = \prod_{j,k^{(1)}} \left(\alpha^{(1)}_{j,k^{(1)}}\right)^{l^{(1)}_{j,k^{(1)}}} \quad , \quad \mathrm{P}(L_2) = \prod_{j,k^{(2)}} \left(\alpha^{(2)}_{j,k^{(2)}}\right)^{l^{(2)}_{j,k^{(2)}}} \quad \text{(S7)}$$

where $\alpha^{(1)}_{j,k^{(1)}}$ and $\alpha^{(2)}_{j,k^{(2)}}$ are the tissue class segmentations of image 1 and image 2, respectively.

1.2 The model

The joint posterior distribution $P(L_1, L_2|I_1, I_2, D, \overline{\gamma})$, can be formulated at every voxel j for tissue class $k^{(1)}$ and $k^{(2)}$ as:

$$p_{j,k^{(1)},k^{(2)}} = \frac{p(y_j^{(1)}, y_j^{(2)}, y_j^{(D)}, l^{(1)}_{j,k^{(1)}} = 1, l^{(2)}_{j,k^{(2)}} = 1, \overline{\gamma})}{\sum\limits_{k^{(1)},k^{(2)}} p(y_j^{(1)}, y_j^{(2)}, y_j^{(D)}, l^{(1)}_{j,k^{(1)}}, l^{(2)}_{j,k^{(2)}}, \overline{\gamma})} \quad \text{(S8)}$$

The sum over all possible tissue classes $k^{(2)}$ of the joint posterior gives us the soft segmentations of the tissue class at time point 1, referred to as $p_{j,k^{(1)}}$. Similarly, the sum over all possible tissue classes $k^{(1)}$ of the joint posterior gives us the soft segmentations of the tissue class at time point 2, referred to as $p_{j,k^{(2)}}$.

$$p_{j,k^{(1)}} = \sum_{k^{(2)}} p_{j,k^{(1)},k^{(2)}} \quad \text{(S9)}$$

$$p_{j,k^{(2)}} = \sum_{k^{(1)}} p_{j,k^{(1)},k^{(2)}} \quad \text{(S10)}$$

In the M-step, a new set of values for model parameter γ is computed by maximising the Q-function. For the Gaussian parameters of the tissue class $k^{(1)}$ we find:

$$\mu_{k^{(1)}}^{(1)} = \frac{\sum_j p_{j,k^{(1)}} y_j^{(1)}}{\sum_j p_{j,k^{(1)}}} \tag{S11}$$

$$\sigma_{k^{(1)}}^{2(1)} = \frac{\sum_j p_{j,k^{(1)}} \left[y_j^{(1)} - \mu_{k^{(1)}}^{(1)}\right]^2}{\sum_j p_{j,k^{(1)}}} \tag{S12}$$

The Gaussian parameters of the tissue class $k^{(2)}$ $\{\mu_{k^{(2)}}^{(2)}, \sigma_{k^{(2)}}^{2(2)}\}$ are derived analogously.

A new set of values for difference image model parameter ζ (see equations-[S4, S5, S6]) is computed by maximising the Q-function having the following closed-form expressions:

$$\mu_{static}^{(D)} = \frac{\sum_j p_{j,k^{(1)}=WM,k^{(2)}=WM} + p_{j,k^{(1)}=lesion,k^{(2)}=lesion}\; y_j^{(D)}}{\sum_j p_{j,k^{(1)}=WM,k^{(2)}=WM} + p_{j,k^{(1)}=lesion,k^{(2)}=lesion}} \tag{S13}$$

$$\sigma_{static}^{2(D)} = \frac{\sum_j p_{j,k^{(1)}=WM,k^{(2)}=WM} + p_{j,k^{(1)}=lesion,k^{(2)}=lesion} \left[y_j^{(D)} - \mu_{static}^{(D)}\right]^2}{\sum_j p_{j,k^{(1)}=WM,k^{(2)}=WM} + p_{j,k^{(1)}=lesion,k^{(2)}=lesion}} \tag{S14}$$

$$\mu_{growth}^{(D)} = \frac{\sum_j p_{j,k^{(1)}=WM,k^{(2)}=lesion}\; y_j^{(D)}}{\sum_j p_{j,k^{(1)}=WM,k^{(2)}=lesion}} \tag{S15}$$

$$\sigma_{growth}^{2(D)} = \frac{\sum_j p_{j,k^{(1)}=WM,k^{(2)}=lesion} \left[y_j^{(D)} - \mu_{growth}^{(D)}\right]^2}{\sum_j p_{j,k^{(1)}=WM,k^{(2)}=lesion}} \tag{S16}$$

$$\mu_{shrinkage}^{(D)} = \frac{\sum_j p_{j,k^{(1)}=lesion,k^{(2)}=WM}\; y_j^{(D)}}{\sum_j p_{j,k^{(1)}=lesion,k^{(2)}=WM}} \tag{S17}$$

$$\sigma^{2(D)}_{shrinkage} = \frac{\sum_j p_{j,k^{(1)}=lesion,k^{(2)}=WM} \left[y_j^{(D)} - \mu^{(D)}_{shrinkage} \right]^2}{\sum_j p_{j,k^{(1)}=lesion,k^{(2)}=WM}} \tag{S18}$$
